# Comparison of flatfeet and normal feet using data of the gait cycle, contact area, and foot pressure

**DOI:** 10.1016/j.dib.2021.106990

**Published:** 2021-03-22

**Authors:** Yuichi Takata, Ryoki Kawamura, Shinji Matsuoka, Hiroshi Hashida, Genta Asano, Kazushi Kimura, Shigenori Miyamoto

**Affiliations:** Department of Physical Therapy, Faculty of Human Science, Hokkaido Bunkyo University

**Keywords:** Flatfeet, Gait cycle, Contact area, Foot plantar pressure

## Abstract

Although the foot is involved in load-bearing and shock absorption, foot pressure (FP), ground contact area (CA), and gait cycle (GC) in flatfeet (FF) have not been examined in detail. We aimed to analyze the influence of FF on FP, CA, and GC. We included 20 and 21 women with FF and normal feet (NF), respectively. A Footscan plantar pressure plate (RsScan International, Belgium) was used to analyze FP, CA, and GC. FP was applied to the unit area of 10 compartments. GC analysis was performed using phase-time measurements by dividing the GC into four phases. In the analysis, FP and CA were compared between the FF and NF groups. A comparison of GC was similarly performed between the two groups.

The data provided in this article will be useful when designing studies on the effect of foot shape on FP, CA, and GC during gait.

## Specifications Table

**Table:** 

Subject	Physical Therapy and Rehabilitation
Specific subject area	Measures plantar pressure while walking. We verified whether plantar pressure changes when differences in the arch structure of the foot affect gait.
Type of data	Table Figure
How data were acquired	RsScan Instruments: RsScan force plate (RS footscan USB 7), which has an area of 40 × 50 cm and 4096 sensors; this device can measure dynamic plantar pressure. Make and model of the instruments used: RsScan International, Belgium
Data format	Raw Analyzed
Parameters for data collection	Data were collected in a quiet room on a leveled surface to measure the sole pressure distribution and walking cycle when walking on level ground.
Description of data collection	The footscan plate was installed 5 m away. The participants were instructed to walk barefoot and step on the RsScan instrument with only one foot.
Data source location	Institution: Hokkaido Bunkyo University City/Town/Region: Eniwa Country: Japan
Data accessibility	Repository name: [Mendeley Data] Direct URL to data: [http://dx.doi.org/10.17632/vv3zgwwpk3.1]

## Value of the Data

• These are important data on the effect of differences in foot type, between flat and healthy feet, on the plantar pressure distribution and walking cycle. They can be used to evaluate the effects of foot shape on gait disturbance.
• These data will be beneficial for researchers in measuring the plantar pressure distribution during walking.
• These data can be used to compare the sole pressure distribution and walking cycle before and after exercise and when using the insole.

## 1 Data Description

The dataset and supplementary files provide relevant details on the plantar pressure distribution and walking cycle, and the data are presented in the tables and figures.

**Fig. 1:**
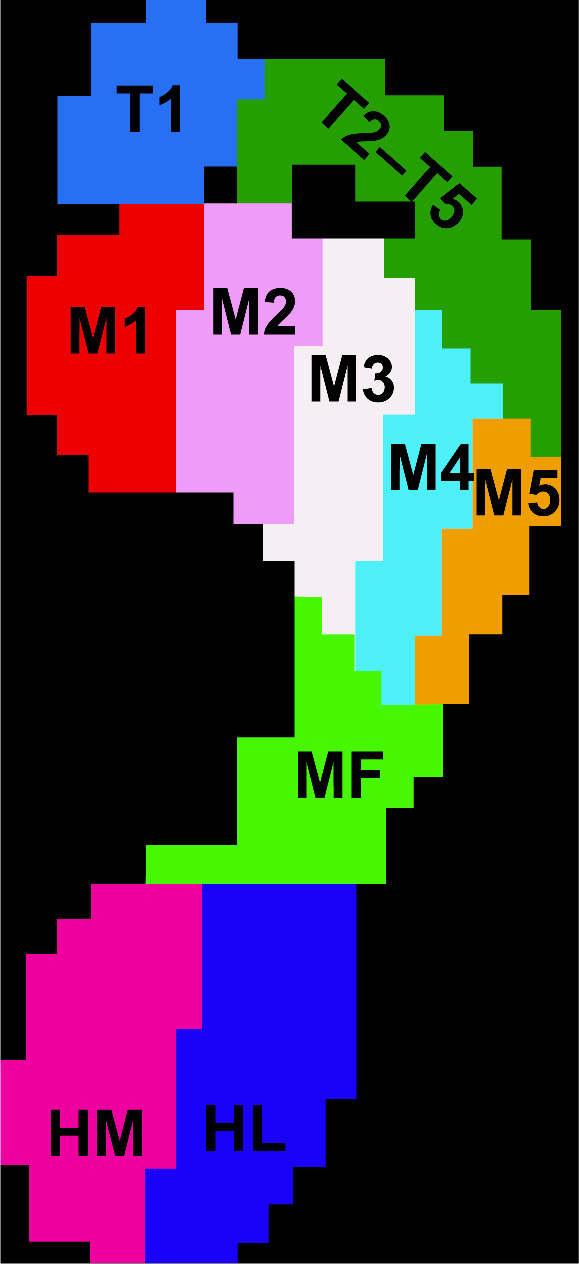
Illustration of the foot, divided into the ten anatomical zones. ■T1: Hallux  ■T2-T5: Toe 2 to toe 5 ■M1: Metatarsal 1  ■M2: Metatarsal 2 ■M3: Metatarsal 3 ■M4: Metatarsal 4 ■M5: Metatarsal 5   ■MF: Midfoot ■HL: Heel Lateral     ■HM: Heel Medial.

**Fig. 2:**
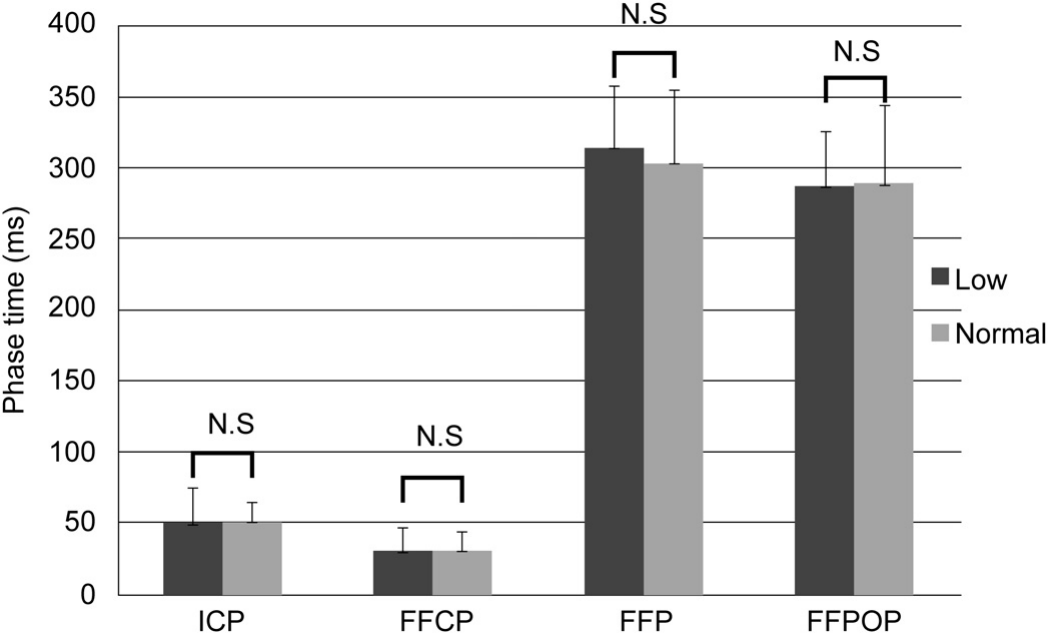
Phase-time comparison of the FF and NF group. FF, flatfeet; NF, normal feet.

The data obtained from participants with flat and healthy feet are presented in Tables 1 and 2 and Figs. 1 and 2. The supplementary file contains the raw data, which are presented as tables in Microsoft Excel.

**Table 1:** Values of MaxP in both groups.

MaxP (N/cm^2^)	FF Group	NF Group	*p*-value
Toe1	6.4 (1.6)*	5.3 (1.6)	0.036
Toe2–5	2.6 (0.1)	2.6 (1.2)	0.923
M1	6.8 (1.9)	6.6 (1.4)	0.705
M2	11.5 (2.1)	11.7 (2.1)	0.802
M3	13.1 (2.7)	12.3 (2.7)	0.362
M4	8.8 (2.0)	8.4 (1.8)	0.483
M5	4.4 (0.7)	4.8 (1.6)	0.283
Midfoot	5.2 (1.3)	4.4 (1.3)	0.094
Heel medial	10.5 (1.7)	10.3 (1.5)	0.685
Heel lateral	9.7 (1.9)	10.1 (1.5)	0.467

MaxP, maximum pressure; FF, flatfeet; NF, normal feet; M1, metatarsal 2; M2, metatarsal 2, M3, metatarsal 3; M4, metatarsal 4; M5, metatarsal 5.

**Table 2:** Values of CA in both groups.

CA (cm^2^)	FF Group	NF Group	*p*-value
Toe1	14.8 (2.8)	15.1 (3.9)	0.785
Toe2–5	18.5 (5.3)	17.1 (4.0)	0.332
M1	14.5 (4.5)	13.8 (2.9)	0.554
M2	11.7 (2.6)	10.9 (1.7)	0.221
M3	9.3 (2.0)	9.7 (3.1)	0.614
M4	9.7 (2.3)	10.1 (2.8)	0.556
M5	13.5 (4.4)	11.5 (2.8)	0.092
Midfoot	49.6 (10.1)*	35.6 (12.7)	0.0003
Heel medial	16.2 (2.9)	16.2 (2.3)	0.956
Heel lateral	14.1 (3.5)	14.2 (1.58)	0.834

Values are expressed as means ± SD.

⁎ *p*<0.05(category). CA, contact area; FF, flatfeet; NF, normal feet; SD, standard deviation; M1, metatarsal 2; M2, metatarsal 2, M3, metatarsal 3; M4, metatarsal 4; M5, metatarsal 5.

The raw data are described [Mendeley Data], and all data for the maximum plantar pressure (MaxP), CA, and phase-time are available. Foot pressure (FP) was defined as the MaxP, and the MaxP and contact area (CA) were measured in the following 10 compartments: Toe1 (T1), Toe2–5 (T2–5), Metatarsal 1 (M1), Metatarsal 2 (M2), Metatarsal 3 (M3), Metatarsal 4 (M4), Metatarsal 5 (M5), Midfoot (MF), Heel medial (HM), and Heel lateral (HL) (Fig. 1).

Participants in the FF and NF groups walked and stepped on the RsScan device, which was placed 5 m away from them, with one foot.

Table 1 shows the results of MaxP per cm^2^ during walking for participants in the FF and NF groups.

A *t*-test was performed for MaxP, for participants in the FF and NF groups, in 10 compartments: T1, T2–5, M1, M2, M3, M4, M5, MF, HM, and HL. In T1, the FF group showed a significantly higher MaxP than the NF group.

Table 2 presents the results of ground CA during walking for participants in the FF and NF groups.

A *t*-test was performed for CA, for participants in the FF and NF groups, in 10 compartments: T1, T2–5, M1, M2, M3, M4, M5, MF, HM, and HL. In MF, the FF group showed a significantly higher CA than the NF group.

Fig. 2 illustrates the comparison of the phase-time (ms) between participants in the FF and NF groups during walking.

The values of initial contact phase (ICP), forefoot contact phase (FFCP), foot flat phase (FFP), and forefoot push-off phase (FFPOP) were 49.7 ± 22.6 ms, 30.3 ± 16.4 ms, 314.4 ± 42.9 ms, and 287.4 ± 38.3 ms, respectively, in the FF group and 50.9 ± 13.2 ms, 30.2 ± 13.3 ms, 304.7 ± 51.8 ms, and 285.4 ± 52.9 ms, respectively, in the NF group. No significant differences were observed in phase-time measurements between the FF and NF groups.

## 2 Experimental Design, Materials and Methods

### 2.1 Participants

Twenty women with FF (mean age, 20.4 [0.8 standard deviation (SD)] years; height, 1.58 [0.03 SD] m; body mass, 53.8 [7.5 SD] kg; foot length, 23.2 [0.7 SD] cm) and 21 women with NF (mean age, 20.5 [0.6 SD] years; height, 1.56 [0.04 SD] m; body mass, 49.1 [4.1 SD] kg; foot length, 23.5 [0.9 SD] cm) were used to acquire these data. The data collected were not case-controlled.

### 2.2 Protocol

The arch index (AI) [1] and functional static navicular drop (FND) [2], [3] were used to evaluate FF. A foot axis was drawn from the center of the heel to the second toe, and the footprint was divided into equal thirds by constructing lines tangential to the foot axis. The AI was calculated as the ratio of the area of the middle third of the footprint to the entire footprint area. AI is the ratio (%) of the area of the middle foot to the foot area excluding the toe.

The AI has received considerable scientific attention and has allowed researchers and clinicians to classify static arch structures as either high (≤0.21), low (≥0.26), or normal (0.21−0.26) [4]. Using this index, we categorized it into the low-arch group and normal-arch group. FND takes a sitting position when the hip and knee joints are flexed 90° The foot was considered flat if the difference between the height of the scaphoid bone from the floor at that time and the height of the scaphoid bone when standing with 50% load on both lower limbs was ≥10 mm [5], [6].

RsScan was used for the measurement. The specifications of RsScan are as follows: Dimensions: 578 × 418 × 12 mm; Effective measuring range: 488 × 325 mm; Number of sensors: 4096; Sensor size: 7.62 × 5.08 mm; Measurement pressure range: 1–127 N/cm^2^; Resolution: 8 bits. The participants were instructed to walk barefoot and step on the RsScan on only one foot for a total of three sets. MaxP areas of the foot were measured during gait at T1, T2–5, M1, M2, M3, M4, M5, MF, HM, and HL and was applied to the unit area of 10 foot regions. Similarly, the pressure applied to the CA was measured. Phase-time measurements of the walking time (ms) were obtained by dividing the walking cycle into ICP, FFCP, FFP, and FFPOP. The values (%) were obtained by dividing each phase time by one GC. ICP was defined as the period from the first heel contact to the first metatarsal contact, and FCP was defined as the period from the first metatarsal contact to the contact of all (M1~M5) metatarsals. While FFP was defined as the period from the contact of all metatarsals until the heel (HM, HL) left the plate, FFPOP was defined as the period from the last heel contact to the last foot contact.

ICP and FFCP correspond to the loading response, FFP corresponds to the mid stance, and FFPOP corresponds to the terminal stance and pre swing [7].

In the analysis, MaxP and CA were compared between the low and normal arch groups, and the time required for ICP, FFCP, FFP, and FFPOP was similarly compared between the two groups. F-test, Student's *t*-test, and Welch's *t*-test were performed on normally distributed data, and the Mann–Whitney U test was conducted on abnormally distributed data. A *p*-value <0.05 was considered statistically significant.

## Ethics Statement

The ethics committee of Hokkaido Bunkyo University approved all study protocols, and each participant provided written informed consent prior to enrollment (number 02008).

## CRediT Author Statement

**Yuichi Takata:** Conceptualization, Methodology; **Shinji Matsuoka:** Statistical analysis; **Genta Asano:** Software; **Ryoki Kawamura:** Investigation; **Kazushi Kimura, Shigenori Miyamoto:** Supervision; **Yuichi Takata, Hiroshi Hashida:** Writing- Reviewing and Editing.

## Declaration of Competing Interest

The authors declare that they have no known competing financial interests or personal relationships, which have or could be perceived to have influenced the work reported in this article.

## Appendix Supplementary materials

Image, application 1

Image, application 2
