# Supplementary material for: Comparison of flatfeet and normal feet using data of the gait cycle, contact area, and foot pressure
Source: Data Brief. 2021 Mar 22;36:106990. doi: 10.1016/j.dib.2021.106990 (PMC8055551; doi:10.1016/j.dib.2021.106990)
Supplement: Supplementary file 2 [file mmc2.pdf]

System specifications

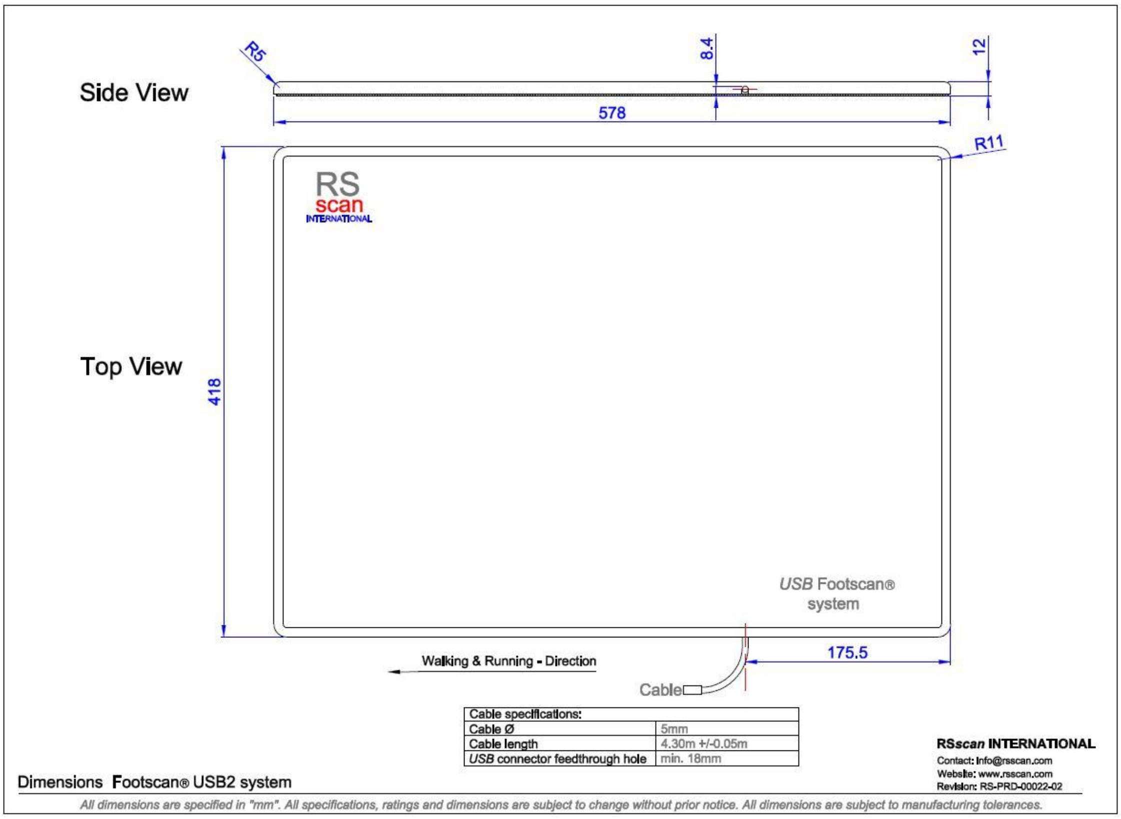

**Dimensions (length x width x height):**	578 mm x 418 mm x 12 mm
**Weight:**	4.2 Kg
**Number of sensors:**	4096 (arranged in a 64 x 64 matrix)
**Sensor dimensions:**	7.62 mm x 5.08 mm
**Active sensor area:**	488 mm x 325 mm
**Sensor technology:**	resistive
**Pressure range:**	1 – 127 N/cm²
**Data acquisition frequency:**	150-300 Hz
**Resolution:**	8 bits
**Operating temperature range:**	+15 °C to +30 °C
**Storage temperature range:**	+0 °C to +40 °C
**Relative humidity:**	20% to 80% non-condensing
**Connection to PC:**	USB 2.0

**Plate cable length:**	4300mm +/- 50mm (integrated cable)
**Power consumption:**	5V @ 500mA maximum
**Protection class:**	I
**IP code:**	IP40

Symbols

The following symbols may appear on the product, packaging or documentation:

	Tip or note.
	Information.
	Caution, read these instructions carefully.<br>If used on the product label: consult accompanying documentation.
	Marking according to the European directive 2002/96/EC on Waste Electrical and Electronic Equipment (WEEE).
SN	Serial number.
	Manufacturer & manufacturing date YYYY MM.
RoHS compliant	Compliant with European directive 2002/95/EC on the restriction of the use of certain hazardous substances in electrical and electronic equipment (RoHS).
	Marking for People’s Republic of China pollution control regulation SJ/T11364–2006.
CE	CE mark according to European directive 93/42/EEC (MDD).
	Type B applied parts.
LATEX	This product contains dry natural rubber.
	This way up.
	Fragile.
